# Does the Harvesting Site Influence the Osteogenic Potential of Mesenchymal Stem Cells?

**DOI:** 10.1155/2019/9178436

**Published:** 2019-05-02

**Authors:** Van Thi Nguyen, Irene Tessaro, Antonio Marmotti, Camilla Sirtori, Giuseppe M. Peretti, Laura Mangiavini

**Affiliations:** ^1^IRCCS Istituto Ortopedico Galeazzi, Milan, Italy; ^2^University of Turin, Department of Orthopaedics and Traumatology, Turin, Italy; ^3^University of Turin Molecular Biotechnology Center, Turin, Italy; ^4^University of Insubria, Varese, Italy; ^5^Department of Biomedical Sciences for Health, University of Milan, Milan, Italy

## Abstract

Total hip arthroplasty (THA) represents one of the commonest surgical procedures in the orthopedic field. Osteointegration of the implant with native bone is essential for an optimal result; thus, the quality of the patient's bone surrounding the implant (i.e., the bone stock) is crucial. However, in some cases, the bone stock is insufficient and needs to be improved with autologous grafts rich in multipotent cells (i.e., from the iliac crest, from the head of the femur, or from the subchondral bone harvested from the acetabulum) or allogenic frozen bone. It is not known if the harvesting site may influence the osteogenic potential of these cells. Thus, our aim was to characterize and compare multipotent cells collected from the bone marrow, acetabular subchondral bone, and trabecular bone on the femoral head with a focus on osteogenic differentiation. The cells from three sources had a fibroblast-like phenotype and expressed surface antigens CD73, CD90, and CD105 and are negative to CD11b, CD34, and CD45. Although all these cells could be induced to differentiate into osteoblasts, chondrocytes, and adipocytes, they displayed different differentiation potentials. In osteogenic differentiation condition, the cells from the acetabulum had the lowest accumulation of calcium deposit while the cells originated from the bone marrow and femur created a considerably increased amount of the deposit. These findings were confirmed by quantitative polymerase chain reaction (qPCR). In chondrogenic and adipogenic conditions, bone marrow cells possessed a predominant differential capacity compared with the others, illustrated by high collagen type II expression together with a cartilage-like lacuna structure and the presence of fat-specific markers, respectively. To our knowledge, this is the first study comparing and demonstrating that the progenitor cells obtained from diverse surgical sites in hip replacement procedure share common characteristics of MSC but differ about plasticity and may provide rational for clinical application in cell therapy and bone grafting. The project number L1033 is registered with ClinicalTrials.gov NCT03369457.

## 1. Introduction

Better health care system and nutrition have increasingly improved life quality and expectancy that results in higher number of elderlies who are also easily affected from aging-related problems including musculoskeletal diseases, i.e., osteoarthritis. According to the data published in OECD's report in 2017, the incidence of total hip arthroplasty (THA) rose by 30% in the organization's countries from 2000 to 2005 [1]. In particular, in 2010, the cases admitted to THA in the United States were 2.1 million accounting to 0.83% population [2]. In the 20th century, John Charnley firstly introduced his hip replacement technique; since then, this technique has been continuously improved to overcome the complications as well as to provide longevity of the prosthesis [3]. Its principle consists of the replacement of the damaged femoral head and acetabulum with a metallic prosthesis composed by a stem fused with a metal or ceramic ball playing a role as the femoral head. An artificial socket is placed in the reamed acetabulum. To facilitate smooth movement between the “ball and socket,” a metal, ceramic, or plastic liner is placed among them. For an optimal clinical result, the implant needs to well integrate with the surrounding bone; thus, the quality of bone and the level of the hip joint (bone stock) are crucial in THA surgery. However, in some cases of complex first implants or in cases of revision surgery, the bone stock may be insufficient. Thus, a bone graft may be required to restore the bone stock [4].

Bone autografts are considered the gold standard since they prevent from graft rejection and they provide osteoinduction (osteoblast stimulation, recruitment, and/or differentiation) and osteoconduction (bone growth) [5]; however, the availability of bone autografts is limited in case of big bone defects. Bone allografts, commonly morselized and cancellous bones from femoral heads, are more available but they are decellularized scaffolds (due to the freezing process); moreover, the risk of infectious disease transmission cannot be dismissed [5]. Ceramics, including hydroxyapatite and tricalcium phosphate, are osteoconductive carriers, which are well known as bone substitutes in orthopedic surgery because of safety, effectiveness, and economic costs [6, 7]. A clinical study performed by Gali et al. [8] demonstrated that a mix of autologous bone marrow aspirate and hydroxyapatite increased bone regeneration in patients with maxillo-mandibular osseous defects. In the orthopedic surgery context, *in vivo* study using bone marrow cells and either tricalcium phosphate or hydroxyapatite-coated acetabular cup in rabbits and goat models, respectively, proved that these mixtures could improve osteoinduction and osteointegration of the grafts, decreasing aseptic loosening [9, 10].

However, to our knowledge, there is still no study addressing and comparing MSC obtained from different anatomic sites during THA procedure. Therefore, our target was to isolate and identify multipotent cells from the acetabular subchondral bone, from fragments of femoral cancellous bones and from bone marrow aspirate, which was used as positive control. We took advantage of the capability of MSC to adhere and grow on the plastic support; thus, we used neither density gradient media nor specific device to isolate the cells from the bone marrow [11]. Moreover, we also stimulated the bone chips to release the cells without collagenase pretreatment which may affect cell behavior [12]; thus, the bone fragments were cultured in the presence of fibroblast growth factor-2 (FGF-2), which improves MSC proliferation and differentiation [13, 14]. Next, multipotent cells were evaluated for cell proliferation, colony-forming unit fibroblast (CFU-f), and expression of MSC cell surface antigens by flow cytometric analysis. Finally, *in vitro* differentiation of the cells into the osteogenic, chondrogenic, or adipogenic lineage was assessed by specific histologic staining methods and by qPCR analysis to measure expression of specific messenger RNA (mRNA). Moreover, for osteogenic differentiation, IL1-*β* was added to the osteogenic medium to better understand the cell differentiation into osteoblast in the *in vitro* inflammatory condition.

We demonstrated that the anatomical site influences the multipotent potential of MSC. Our results may give some new *hints* for *in vivo* research and stem cell therapy for bone tissue engineering.

## 2. Materials and Methods

Approval was obtained from the Ethical Committee of IRCCS Istituto Ortopedico Galeazzi.

For patient selection, 9 patients undergoing THA procedure at Galeazzi Orthopedic Institute (Milan, Italy) were enrolled in the study after signing an informed consent.

Inclusion criteria were age between 50 and 80 years old; body mass index (BMI) was between 18 and 30 kg/m^2^; there was no history of metabolic bone disease; there was no history of infectious diseases (HIV, HCV, HBV, or Treponema pallidum) or inflammatory joint disease (i.e., rheumatoid arthritis).

### 2.1. Cell Isolation and Explant Culture

Bone marrow aspirate from the femoral canal, pieces of reamed acetabular subchondral bones, and fragments of femoral cancellous bones were obtained from the patients. Samples were stored at 4°C and processed within 5-6 hours (h) from surgery.

The bone marrow was diluted 1 : 1 with sterile 1x phosphate-buffered saline (PBS) and centrifuged at 1,700g for 10 min at room temperature (RT). The supernatant was carefully removed. This step was repeated twice. Cell pellet was resuspended in PBS, and the first test sample was diluted 1 : 10 volume/volume (*v*/*v*) in PBS (suspension A). From the suspension A, the second test sample was taken and mixed 1 : 1 *v*/*v* in 4% acetic acid (suspension B) and incubated for 1 minute (min) to eliminate red blood cells and white blood cells. From the suspension B, the third test sample was taken and mixed 1 : 1 *v*/*v* in trypan blue to stain dead cells. Nucleated cells were counted and plated at a density of 5 × 10^4^ cells/cm^2^ in complete medium containing alpha minimum essential medium (*α*MEM, Life Technologies), 10% *v*/*v* fetal bovine serum (FBS, Euroclone), and 1% *v*/*v* penicillin/streptomycin/glutamine (P/G/S, Life Technologies) supplemented with 1 ng/ml recombinant human FGF-2 (R&D Systems) on T-175 flasks. The primary cells at passage 0 (P0) were incubated at 37°C and 5% CO_2_ without disturbance for 1 week. Then, medium was changed twice per week. When cells reached subconfluence, they were detached by using 1x trypsin/ethylenediaminetetraacetic acid (trypsin/EDTA, Life Technologies) and passaged up to P2. Acetabular and femoral bone pieces were intensively washed in PBS to remove blood and then fragmented. The minced chips were washed again in PBS and then maintained in complete medium supplemented with 1 ng/ml FGF-2 on 6-well plates. The medium was changed two times every week. When cells released from the pieces reached subconfluence, trypsin/EDTA was added to the wells to trypsinize cells (the bone chips were in contact with trypsin/EDTA as well). The cells were expanded up to P2. The bone pieces were discarded after two times in trypsin/EDTA treatment.

### 2.2. Colony-Forming Unit Fibroblast (CFU-f) Assay

Cells at P1 were plated at a density of 1 × 10^3^ cells/10 cm dish in complete medium supplemented with FGF-2. Each experiment was performed in triplicate, and the medium was changed twice per week. After two weeks, cells were washed with 1x PBS and then fixed in methanol and absolute ethanol (1 : 1 *v*/*v*) for 10 min followed by the coloration in 0.5% g/ml crystal violet solution (solvent was 20% *v*/*v* methanol) (Sigma) for 15 min at room temperature (RT). Then, cells were washed twice with PBS and running tap water to remove the excessive stain. The dishes were air-dried, and only colonies containing more than 50 cells were counted. Results were expressed as average of at least four independent experiments performed on at least four different primary cell cultures + standard error of the mean (SEM) values. Photos of the dishes were taken using the scanner Epson V200 (Epson).

### 2.3. Cell Proliferation and Viability

#### 2.3.1. Crystal Violet Stain

Crystal violet stain was performed to determine cell proliferation in monolayer culture. Cells were plated at a density of 1.5 × 10^3^ cells/well in complete medium consisting of FGF-2 in quintuplicate on 96-well plates. At each time point, cells were washed once with PBS, fixed and stained with 50 *μ*l colorant solution (0.75% g/ml crystal violet (Sigma), 0.35% g/ml NaCl, 32.3% *v*/*v* absolute ethanol, and 35% *v*/*v* formalin 10%) for 20 min at RT, and finally washed 5 times with water and air-dried. Photos were taken by using the bright field microscope (Leica). 100 *μ*l of eluent solution (50% *v*/*v* absolute ethanol and 1% *v*/*v* acetic acid) was added to each well, and the dye absorbance was measured at 570 nm wavelength using the microplate reader model 680 (Bio-Rad). The experiment was repeated at least three times on at least three different primary cell cultures.

#### 2.3.2. MTT (3-(4,5-dimethylthiazol-2-yl)-2,5-diphenyltetrazolium bromide) Stain

MTT stain was used to investigate cell viability and proliferation cultured as pellet in suspension culture (see 2.5.2). Cultures were performed in 15 ml falcon tubes at a density of 5 × 10^4^ cells/0.5 ml/tube up to 21 days. The experiment was repeated at least three times in triplicate on at least three different primary cell cultures. For the stain, tubes were centrifuged at 1,700g for 5 min, pellets were washed once with PBS, and 0.5 ml serum-free medium supplemented with 25 *μ*l of MTT (Sigma) of 5 mg/ml stock solution was added to the tubes. Cells were incubated for 1 h in the dark. Then, pellets were washed twice with PBS. 1 ml of absolute ethanol was added to each tube; tubes were vortexed for 1 min to solubilize the purple formazan. The absorbance of the colorant was measured at 570/670 nm wavelength by using spectrophotometer Ultrospec 2100 (Amersham Biosciences).

### 2.4. Flow Cytometric Analysis

When cells reached 80% of confluence, they were detached by using trypsin/EDTA and resuspended in stain buffer composed of 1x PBS and 3% *v*/*v* FBS that block nonspecific antigens. The cell suspension was divided into polystyrene round bottom tubes at a density of 10^5^ cells/tube. The incubation was performed at 4°C for 20 min in the dark using monoclonal antibodies against CD11b (clone ICRF44), CD34 (clone 581), CD45 (clone HI30), CD73 (clone AD2), CD90 (clone 5E10), and CD105 (clone 266). For automatic compensation, anti-mouse Ig, *κ*/negative control compensation particle set was used. All the reagents were purchased from BD Biosciences. Furthermore, to better interpret the results and gating, we also used the fluorescence minus one (FMO) method, where tubes contain all the antibodies except the one which is being measured. After staining, the cells were washed with PBS, resuspended in 0.5 ml stain buffer, and run through flow cytometer BD FACS Canto II (BD Biosciences). Data were analyzed by software FlowJo.

### 2.5. In Vitro Differentiation

#### 2.5.1. Adipogenic Differentiation

To induce adipogenic differentiation, cells were cultured in complete medium supplemented with FGF-2 at 5 × 10^4^ cells/well on 24-well plates or 3 × 10^5^/well on 6-well plates for Oil Red O stain and qPCR, respectively. For Oil Red O stain, the experiment was performed in triplicate. When the cells were confluent, the medium was changed to adipogenic medium which is complete medium composed of 50 *μ*g/ml ascorbic acid, 10^−7^ M dexamethasone, 5 ng/ml insulin, 0.5 mM 3-isobutyl-1-methyl xanthine, and 0.1 mM indomethacin. All the reagents were bought from Sigma. The complete medium was used as control. The induction media were freshly prepared and changed two times per week. The induction lasted for 3 weeks.

#### 2.5.2. Chondrogenic Differentiation

Induction of chondrogenic differentiation was performed as previously described [15] with little modification. In this study, cells were suspended in 15 ml falcon tubes in complete medium containing 50 *μ*g/ml ascorbic acid (Sigma), 10^−7^ M dexamethasone (Sigma), and 10 ng/ml recombinant human transforming growth factor-*β*1 (TGF-*β*1, R&D Systems) at a density of 5 × 10^5^ cells/0.5 ml/tube for immunohistochemistry and toluidine blue staining, 5 × 10^4^ cells/0.5 ml/tube for MTT assay, and 3 × 10^5^ cells/0.5 ml/tube for qPCR. The media were changed twice per week. The cultures were maintained up to 21 days.

#### 2.5.3. Osteogenic Differentiation

To stimulate osteogenic differentiation, cells were firstly cultured in complete medium supplemented with FGF-2 at 5 × 10^4^ cells/well on 24-well plates or 3 × 10^5^ cells/well on 6-well plates for Alizarin Red S stain and qPCR, respectively. For Alizarin Red S stain, the experiment was carried out in triplicate. When the cells reached confluence, the medium was switched to osteogenic medium (OM) which is complete medium containing 50 *μ*g/ml ascorbic acid (Sigma), 10^−7^ M dexamethasone (Sigma), and 10 mM *β* glycerolphosphate (Merck). Moreover, 50 pg/ml IL1-*β* (R&D Systems) was also added to the OM. The concentration of IL1-*β* used for this experiment procedure was inferred from literature data [16–18]. The complete medium was used as control. OM and OM supplemented with IL1-*β* were prepared freshly and added to the cells twice per week. The differentiation was prolonged for 3 weeks.

### 2.6. Cell Staining and Immunohistochemistry

#### 2.6.1. Oil Red O Stain

Oil Red O coloration was used to stain the lipid droplets formed by adipocytes. The working staining solution was prepared freshly (1-24 h) before the experiment by mixing 6 : 4 *v*/*v* of 0.5% Oil Red O solution (Sigma) and distilled water, and then the solution was filtered to discard the precipitates. The cells were washed once with PBS, fixed in 10% formalin for 10 min, and washed again with PBS to remove formalin. PBS was completely removed followed by coloration in the working solution for 10 min at RT. Then, cells were washed once in 60% isopropanol solution for 30 seconds (s) and rinsed three times with distilled water. Photos of the cells with stained droplets were immediately taken by using the bright field microscope (Leica). Then, the water was removed, and the stained droplets were dissolved in isopropanol solution (1 ml/well). Dye absorbance was measured at 510 nm wavelength using spectrophotometer Ultrospec 2100.

#### 2.6.2. Toluidine Blue O Stain

Toluidine blue O stain was used to observe the cartilage matrix. The colorant stains nuclei as blue and proteoglycans and glycosaminoglycans as light blue to violet. Cell pellets were washed twice in PBS, fixed in 10% formalin for 15 min at RT, then included in paraffin, and sliced into 5 *μ*m sections on the SuperFrost Plus adhesion slides (Thermo Fisher Scientific) which were previously coated with poly-L-lysine (Sigma) to prevent section from detachment.

For the coloration, slides were deparaffinized and dipped in the 0.2% *w*/*v* toluidine blue O solution (the powder was provided by Serva, Germany) for 5 min at RT followed by rinsing in distilled water twice, each time for 5 min and mounted with DPX mounting medium.

#### 2.6.3. Immunohistochemistry

The slides were prepared as mentioned above. After deparaffinization, antigen retrieval process was performed by immersing the slides in boiling citrate buffer (pH 6) and cooled down at RT for 20 min. Next, sections were permeabilized in 0.2% Triton in PBS for 10 min at RT, washed three times with PBS, each for 5 min, and incubated in 4% hydrogen peroxide for 30 min at RT to block the endogenous peroxidase activity. Then, slides were incubated in hyaluronidase type II (Sigma) at a concentration of 1 mg/ml in PBS (pH 6) for 30 min at 37°C to break down the matrix and blocked in 10% FBS for 1 h at RT followed by incubating with primary antibodies anti-collagen type I (ab138492, Abcam) and anti-collagen type II (ab34712, Abcam) for 16 h at 4°C. After washing with PBS, the sections were incubated with anti-rabbit biotinylated antibody (1 : 500, Dako) for 30 min at RT, washed in PBS, incubated in streptavidin peroxidase (Dako) for 30 min at RT, then stained with 3,3′-diamino-benzidine (DAB, Dako) for 3 min, counterstained in Mayer's hematoxylin for 1 min, submerged in distilled water containing some drops of 1 M NH_4_OH (Sigma) for bluing, and mounted with DPX mounting medium. Photos were taken by using a light microscope (DM5000 B, Leica).

#### 2.6.4. Alizarin Red S Stain

To observe the mineralization of the induced cells in osteogenic differentiation, Alizarin Red S stain was used. Briefly, cells were washed once with PBS, fixed in 10% formalin for 10 min, washed twice with PBS, stained in 2% *w*/*v* Alizarin Red S solution pH 4.2 (the powder was purchased from Sigma) for 10 min at RT, and washed with distilled water. The plates were air-dried later. To dissolve the calcium deposits, 1 ml of elution solution containing 20% methanol and 10% acetic acid was added to each well and incubated for 30 min at RT with gentle pipetting and shaking. The dye's absorbance was measured at 450 nm wavelength. The experiment was repeated at least six times on at least five different primary cell cultures.

### 2.7. qPCR

qPCR was performed to examine the expression of mRNA extracted from the differentiated cells. Total RNA was isolated by using RNeasy Mini Kit (Qiagen). NanoDrop 8000 (Thermo Fisher Scientific) was utilized to assess RNA quality and purity. If required, RNA was precipitated by mixing with 1 : 3 *v*/*v* cold absolute ethanol and 1 : 0.1 *v*/*v* 3 M sodium acetate and incubating for 16 h at −80°C. 1 *μ*g of RNA was reversely transcribed by using ImProm II reverse Transcription System (Promega) on thermal cycler (Euroclone). Amplification of cDNA was carried out using PowerUp SYBR master mix (Thermo Fisher Scientific) and primers were purchased from Eurofins (Germany) on 7500 Fast Realtime PCR System (Applied Biosystems). The sequences of primers are listed in Table 1. The reaction plates were run in three stages: holding stage starts at 50°C for 20 s and then 95°C for 10 min, cycling stage was repeated for 40 cycles where the reactions were remained at 95°C for 15 s and then 60°C for 1 m, and finally in the melt curve stage, the plates were set at 95°C for 15 s, followed by 60°C for 1 m, 95°C for 30 s, and 60°C for 15 s. Relative gene expression was done according to comparative method [19] where data were presented as 2^−ΔΔCt^ or 2^−ΔCt^ with *∆*Ct = Ct (gene of interest)-Ct (housekeeping gene) and *∆∆*Ct = *∆*Ct at day *n*-∆Ct at day 0, *n* = number of days of differentiation.

### 2.8. Statistical Analysis

All data are presented as means and standard error of the mean (SEM). Statistical analysis was performed by using unpaired *t*-test online application of the GraphPad software (https://www.graphpad.com/quickcalcs/ttest1); for analysis of cell proliferation in adherent culture condition, the nonparametric Mann–Whitney *U* test with two-tailed hypothesis was applied (https://www.socscistatistics.com/tests/mannwhitney/). Analysis is considered significant if *p* value is <0.05.

## 3. Results

### 3.1. Morphology, Proliferation, and Phenotypic Characterization of the Cells in Adherent Conditions

**Figure 1:**
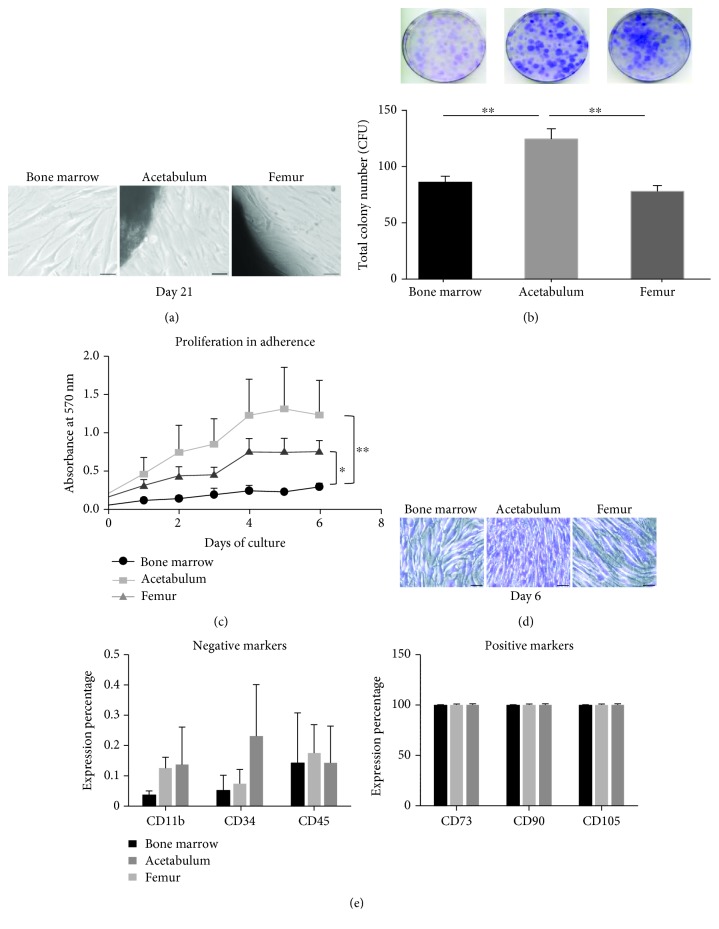
Cell morphology, proliferation, and marker expression in monolayer culture. (a) Morphology of the cells in culture at day 21. The middle and the right photos show the cells released from the bone chips (on the top left and bottom left corner, respectively) and expanding, scale bar 50 *μ*m. (b) Cell colonies stained with crystal violet solution after plating 14 days showing a 1.5- and 1.6-fold increase in the cells derived from the acetabulum compared with the cells from the bone marrow and femur, respectively. Data are shown by the average + SEM of at least four independent experiments performed in triplicate from at least four different primary cultures, ^∗∗^*p* < 0.006. (c) Proliferation curve of the cells in adherent culture determined by measuring absorbance of the cells stained with crystal violet at 570 nm wavelength. Results are the average + SEM of at least three experiments done in quintuplicate from at least three different cell cultures (^∗^*p* = 0.0146, ^∗∗^*p* = 0.0076). (d) Morphology of cells stained with crystal violet in culture at day 6, scale bar 50 *μ*m. (e) Charts showing cell marker expression analyzed by flow cytometry. The expression percentage of the antigens is the average + SEM from three independent experiments of three different primary cell cultures (*p* > 0.05).

The first criteria to identify MSC is their adherence to a plastic support [20]. Taking advantage of this basic MSC feature, we used a very simple method to isolate MSC from the bone marrow (BMC) and from femoral (FC) and acetabular (AC) bone chips in the explant cultures. Bone marrow samples were washed and left without disturbance; after one week, the adherent cells were observed while for explant culture, after approximately two weeks, the first fibroblast-like cells were released from the chips (Figure 1(a)). Moreover, cells also grew to form colonies starting from low plating density as demonstrated in Figure 1(b). After plating cells for 14 days, AC showed a striking capacity of colony formation compared with the cells from the bone marrow and femur (1.5- and 1.6-fold increase, respectively). Cell growth was also investigated by staining the cells with crystal violet solution following time course.  Figure 1(c) shows a significant proliferation of AC and FC compared with BMC (*p* = 0.0076 and *p* = 0.0146, respectively). The difference in cell proliferation was also illustrated in Figure 1(d): day 6 of culture cells from the bone marrow still did not reach confluence. In contrast, AC and FC were tight, and in particular, AC appeared much smaller than the others in spite of the same seeding density inferring a faster growth.

To more comprehensively characterize the cell population from the three sources, we used flow cytometry to evaluate expression profile of markers (Figure 1(e)). BMC possessed a lower percentage of markers for hematopoietic cells and endothelial cells such as CD11b, CD34, and CD45 compared with AC and FC, without significant difference. Moreover, there was no significant variation in expression of the cell surface markers commonly used to define MSC such as CD73, CD90, and CD105.

### 3.2. In Vitro Adipogenic Differentiation

**Figure 2:**
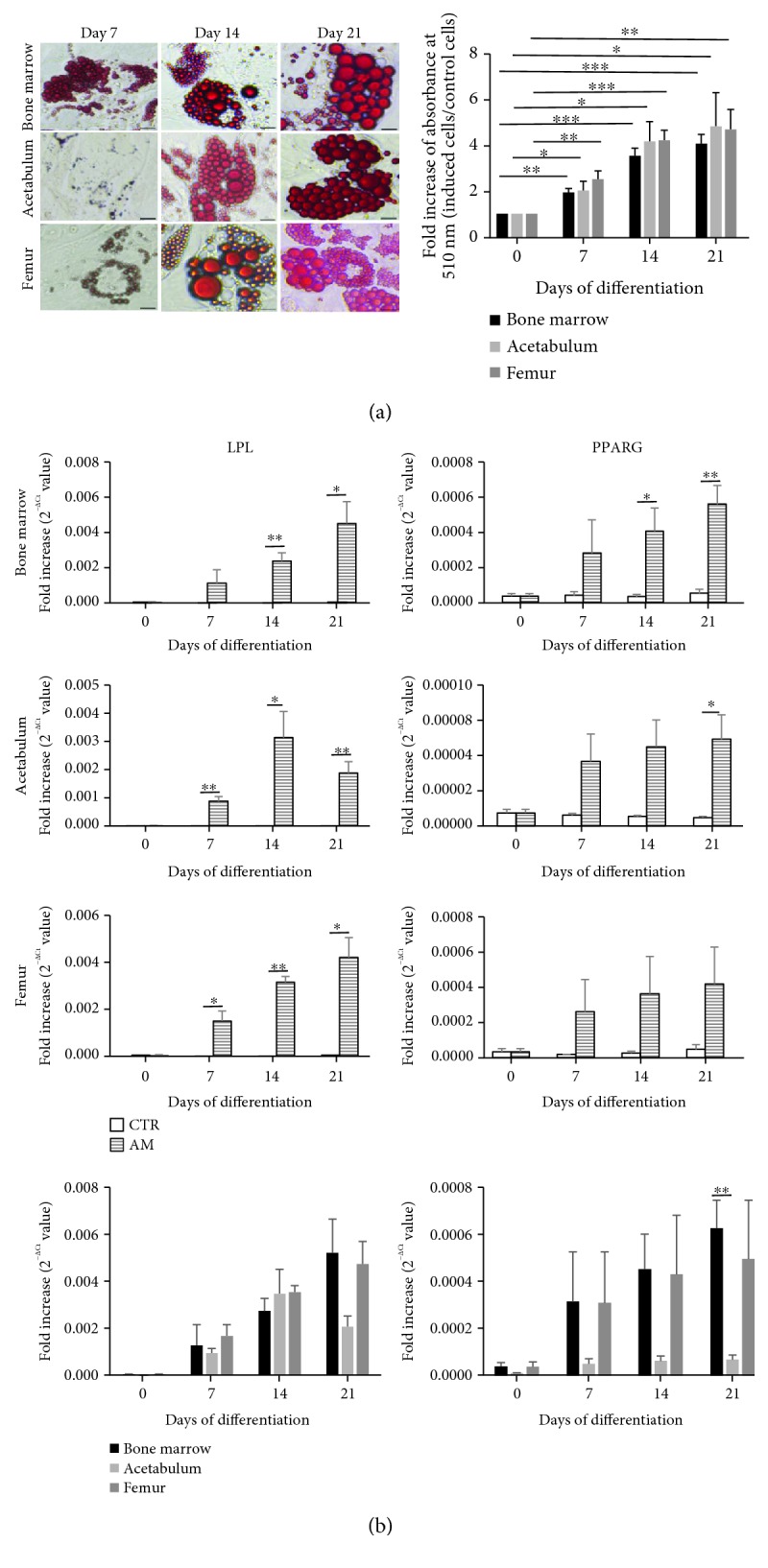
Adipogenic differentiation. Confluent cells were induced in adipogenic medium (AM) for three weeks to differentiate into adipocytes; the control (CTR) cells were maintained in complete medium. (a) Oil Red O stain. Cells were stained with Oil Red O solution, which marks fat droplets into red color. Photos of the CTR cells are not shown, scale bar 50 *μ*m. Quantification was determined by measuring the absorbance of the stained cells at 510 nm (*n* = 5, ^∗^*p* < 0.05, ^∗∗^*p* ≤ 0.003, ^∗∗∗^*p* ≤ 0.0003). (b) qPCR results. Expression of the adipocytic marker genes LPL and PPARG of the cells cultured in CTR and AM conditions. Data are presented as 2^−ΔCt^ + SEM value. The lowest charts show the expression comparison of these markers among the cells originated from all three sources (*n* = 3, ^∗^*p* < 0.05, ^∗∗^*p* ≤ 0.0092).

To reveal the *in vitro* formation of adipose tissue, the cells were exposed to adipogenic medium for 21 days. After 7 days, the small fat vacuoles were detected by Oil Red O staining, and their size and density increased over time as shown in Figure 2(a). Quantification of dyed fat droplets was conducted by measuring the absorbance at 510 nm, and results were presented as fold increase in absorbance between the induced cells and the control cells. The chart in Figure 2(a) proved that even at early treatment time, there is still significant increase in adipogenic capacity of the cells in respect of day 0. The cells from three sources have similar expression of fat droplets. Therefore, qPCR was performed to further evaluate gene expression of two adipogenic markers: LPL and PPARG (Figure 2(b)). Interestingly, all three cell types showed a higher LPL expression than PPARG. Both BMC and FC showed an increase expression of LPL over the time, with a significant difference between the induced cells and control cells starting from day 14 (BMC) to day 7 (FC), and while LPL expression in AC reached a significant difference at day 7, it reached peak at day 14 and then decreased at day 21. PPARG expression in induced FC over time was higher compared with that in the control cells but not statistically significant.

We also compared LPL and PPARG among the three cell types. BMC showed a higher expression of both LPL and PPARG at the latest time point, as shown in the last two charts of Figure 2(b). Moreover, the difference in PPARG expression is statistically significant after 21 days between BMC and AC.

### 3.3. In Vitro Chondrogenic Differentiation

**Figure 3:**
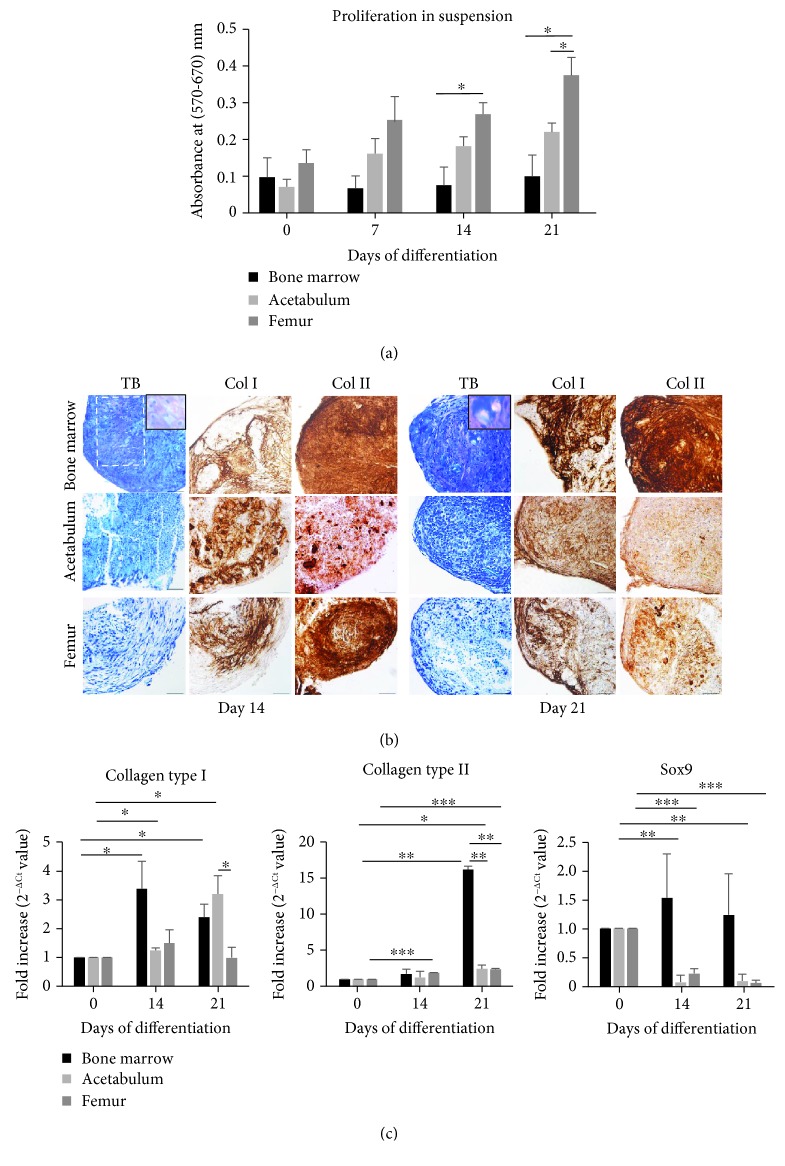
Chondrogenic differentiation in suspension condition. Cells were induced in chondrogenic medium in suspension condition to form pellets up to 21 days and then analyzed. (a) Proliferation of the cells determined by MTT assay. Absorbance of the stained cells was measured at 570 nm and 670 nm (reference wavelength). Results are the average + SEM value of at least three experiments performed in triplicate from at least three independent cell cultures. (b) Photos of toluidine blue O (TB) stain and immunohistochemistry for collagen type I (Col I) and collagen type II (Col II). Toluidine blue stains the matrix from light blue to violet and dyes the nuclei to dark blue. The white dash line covers the most violet zone on the photo showing the coloration of the more intensified cartilage matrix. The insets better illustrate cartilaginous matrix where chondrocytes are trapped in the lacunae. The brown color indicates the expression of collagen type I and collagen type II. Scale bar: insets 5 *μ*m, others 25 *μ*m. (c) qPCR results. Expression of the cartilage markers collagen type I, collagen type II, and Sox9. Data are presented as 2^−ΔΔCt^ + SEM value (*n* = 3, ^∗^*p* < 0.05, ^∗∗^*p* ≤ 0.0025, ^∗∗∗^*p* ≤ 0.0007).

To unravel the chondrogenic capacity of the cells, we used the pellet culture method in three-dimensional suspension condition, which more closely mimics the *in vivo* cartilaginous microenvironment. First, we studied cell proliferation in two-dimensional adherent conditions (Figure 1(c)). The evaluation of cell viability and/or proliferation in suspension conditions is also recommended to predict cell behavior since differentiation in three-dimensional environment may interfere with proliferation.  Figure 3(a) represents cell proliferation in suspension: BMC maintained almost unchanged growth while AC and FC continuously proliferated. At day 21, FC displayed an impressive growth capacity compared with BMC and AC (3.76- and 1.69-fold increase, respectively).

We then proceeded with the analysis of the cartilaginous phenotype by analyzing glycosaminoglycan deposition and the expression of cartilaginous markers, namely, collagen type II and Sox9. Unfortunately, *in vitro* chondrogenesis often produces fibrocartilage characterized by the expression of collagen type I [22]; therefore, in this study, we also evaluated its expression. Toluidine blue O stain was used to observe the formation of extracellular matrix of the pellet.  Figure 3(b) shows that all of the cells were able to form pellets, but the intensity of the stain changed among the groups. BMC pellets exhibited a localized violet area at day 14 suggesting an initial formation of the *in vitro* cartilage matrix; after 21 days of treatment, the violet zone expanded and covered most of the section. More interestingly, we also observed the cartilage typical lacuna structure where the chondrocytes are embedded (insets in Figure 3(b)). On the other hand, AC and FC only showed light blue coloration of the matrix without any lacuna structure even at the longest time point. Production of collagen type I and collagen type II was analyzed by immunohistochemistry (Figure 3(b)). At day 14, BMC and FC expressed a stronger signal for collagen type II than AC; however, at day 21, FC showed much less intensified signal for collagen type II than BMC. Collagen type I was unexpectedly present on all cell pellets suggesting the unselective differentiation despite a long culture in three-dimensional conditions.

We also investigated gene expression for collagen type I, collagen type II, and chondrogenic specific marker Sox9 by qPCR (Figure 3(c)). At day 21 of culture, collagen type I expression decreased in BMC and FC compared with the earlier time point; that result was not found in AC, though a significant difference was not detected. More importantly, after 21 days of induction, collagen type II expression in BMC cells reached the peak and was significantly higher compared to the AC and FC cell sources (6.58- and 6.78-fold increase, respectively). BMC showed an increase in Sox9 expression but not in a significant manner, while in AC and FC, the expression markedly decreased with time. These findings confirmed the abovementioned histological observation.

### 3.4. In Vitro Osteogenic Differentiation

**Figure 4:**
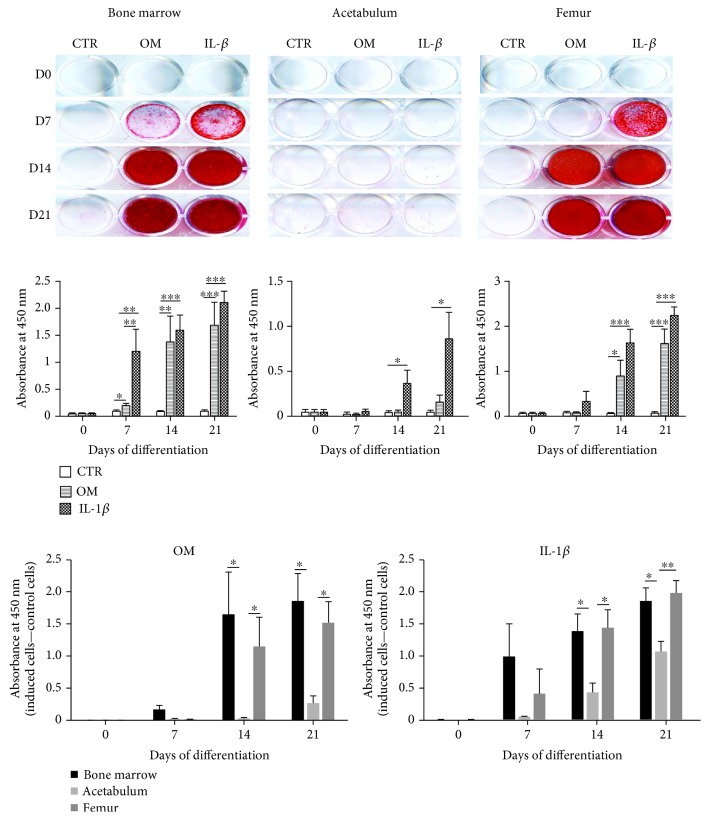
Osteogenic differentiation determined by Alizarin Red S coloration. Confluent cells were cultured and induced in osteogenic medium (OM) or OM supplemented with 50 pg/ml IL-1*β* (IL-1*β*) up to 21 days. The control (CTR) cells were maintained in complete medium. Alizarin Red S solution was used to recognize calcium deposits. Quantification was performed by measuring the absorbance of the stained deposits at 450 nm wavelength. The lowest charts show the comparison in quantification among the cells originated from all three sources in both OM and IL-1*β* conditions. Data are the average + SEM value of at least six independent experiments carried out in triplicate from at least six different cell cultures (^∗^*p* < 0.05, ^∗∗^*p* < 0.009, ^∗∗∗^*p* < 0.0008).

The confluent cells were induced to differentiate into osteoblasts in OM or OM supplemented with IL-1*β* conditions. The Alizarin Red stain in Figure 4 revealed that even under long experimental time, AC are the most unresponsive cells to both conditions OM and IL-1*β* while BMC and FC started to react to the induction from day 7. In both OM and IL-1*β* conditions, starting from day 14, AC had the lowest accumulation of calcium with respect to the other two cell sources (the last two charts in Figure 4). In OM condition, BMC showed the highest level of mineral deposits, while in IL-1*β* condition from day 14, FC released the most elevated amount of deposits; although in both conditions, no significant difference was measured between these two groups. The staining also verified that the presence of IL-1*β* at appropriate concentration did not interfere with the mineralization of the cells in osteogenic differentiation medium.

**Figure 5:**
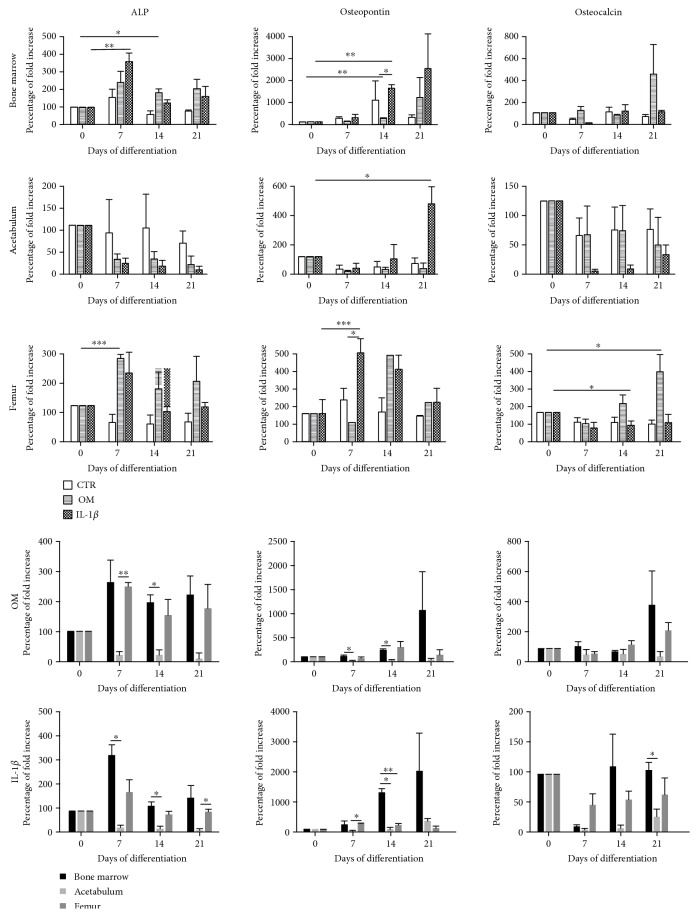
Osteogenic differentiation analyzed by qPCR. Expression of the bone markers ALP (early-stage differentiation), osteopontin (middle-stage differentiation), and osteocalcin (late-stage differentiation). The charts on the last two rows show the comparison in marker expression of the cells derived from three sources in both conditions OM and IL-1*β*. Data are shown as percentage of fold increase 2^−ΔΔCt^ + SEM value (*n* = 3, ^∗^*p* < 0.05, ^∗∗^*p* ≤ 0.0061, ^∗∗∗^*p* ≤ 0.0008).

We also performed qPCR (Figure 5) to fully investigate gene expression of osteogenic markers such as ALP (early-stage osteogenesis), osteopontin (middle-stage osteogenesis), and osteocalcin (late-stage osteogenesis) [23]. BMC and FC when exposed to OM or IL-1*β* conditions highly expressed ALP gene at the earliest time point (day 7) and then the expression decreased over time.

In AC, ALP expression at the different time points was even lower compared with day 0 but without significant difference. In FC, a significant upregulation in osteopontin gene expression was noticed at day 7, whereas in BMC and AC, a significant difference was observed later at day 14 and day 21, respectively. Moreover, a significant upregulation in osteocalcin gene expression was detected only in FC at days 14 and 21 when compared with day 0. As mentioned previously, we compared cells cultured in a standard OM medium with cells cultured in OM supplemented with 50 pg/ml IL-1*β*, to test its possible role in increasing osteogenesis. By Alizarin staining, we did not observe a sufficient difference in mineralization between two conditions OM and IL-1*β* (Figure 4), whereas by qPCR, we noted that IL-1*β* when used at 50 pg/ml concentration in osteogenic medium may increase osteopontin expression in BMC and FC at day 14 and day 7, respectively.

The charts on the last two rows in Figure 5 compared ALP, osteopontin, and osteocalcin gene expression of the cells in each condition. In OM condition, BMC and FC showed a significant upregulation in ALP gene expression when compared with AC; in particular, in FC, the significant difference was present at day 7. Osteopontin gene expression was significantly upregulated in BMC when compared with AC (*p* = 0.0403 at day 7 and *p* = 0.0111 at day 14). Osteocalcin gene expression did not significantly change among the three different cell sources. Finally, in OM standard conditions, BMC generally showed an upregulation of all the three markers when compared with FC, though without a significant difference.

In OM+IL-1*β* condition, BMC and FC also had significantly upregulation in ALP and osteopontin gene expression compared with AC. Moreover, we observed only a significant difference between BMC and FC in osteopontin gene expression at day 14; in particular, the bone marrow cells yielded a higher amount of osteopontin (*p* = 0.0023). All these findings suggest that the osteogenic potential of the bone marrow-derived cells is quite competitive compared with that of progenitor cells released from femoral bone fragments.

## 4. Discussion

The heterogeneity of MSCs, which not only differ from donor to donor but also vary depending on the source of tissues, has been discussed in previous works [24–26]. Therefore, the search and selection of the best source of MSCs for a specific clinical application is required. In particular, to our knowledge, little work has been performed to compare the multipotency of cells obtained from the surgical sites during hip arthroplasty. In this study, we isolated and compared cells derived from different tissues in hip replacement procedure: the bone marrow collected from the femoral canal (as positive control, BMC), pieces of acetabular subchondral bone (AC), and fragments of trabecular bone from the femoral neck and head (FC). The aim of our work was to analyze potential differences between cell sources that may be applied for new techniques of bone stock restoration. Our study proves that three types of cells share MSC identity, but they show differences in cell lineage differentiation that may indicate variations in cell commitment.

CFU-f assay has been considered the gold standard to evaluate the stemness of progenitor cells [27, 28]; namely, starting from a scarce plating number, the progenitor cells were able to attach the plastic culture dish, proliferate, and form colonies. Cells released from pieces of the acetabulum showed a great ability of colony formation compared with the cells from the bone marrow and femur. This finding is consistent with the proliferation assay in adherent culture demonstrating the striking growth capacity of AC. We did not observe a significant difference in cell marker expression, as all of the cell types were negative for hematopoietic markers (CD11b, CD34, and CD45) and positive for MSC markers (CD73, CD90, and CD105).

The iliac crest bone marrow has been accepted as “gold standard” source for MSC isolation [29, 30] because of easy access and high content of red marrow. The bone marrow obtained from femoral canal is occupied by red marrow and also yellow marrow composed of adipocytes. Even though all of the cells isolated from the three sources formed visible fat droplets, the presence of primitive adipocytes could be an explanation for the predominant expression of fat gene markers LPL and PPARG of BMC compared to AC and FC when exposed to adipogenic medium. The concept of the bone marrow “stem cell niche”, where the bone marrow represents the microenvironment where MSCs and hematopoietic stem cells (HSCs) reside, is well known [31–34]. In the niche, the MSC is a main player [32], which interacts, orchestrates, and maintains the homeostasis of the microenvironment. Thus, MSCs are more naïve and are able to quickly respond to the microenvironment when differentiation is needed to replace dead or damaged cells. Indeed, under a chondrogenic stimulus, BMC were able to form the *in vitro* lacunae with strong expression of collagen type II and high quantity of glycosaminoglycans, whereas AC and in particular FC displayed an inferior chondrogenic potential as shown by histology and by gene expression. Therefore, both AC and FC progenitor cells may differentiate into chondrocytes if properly stimulated, but their intrinsic chondrogenic potential is inferior compared with bone marrow-derived cells. In osteogenic conditions, we noticed significant differences between the cell sources: quantification of Alizarin Red S staining demonstrated that both BMC and FC have a significantly higher capacity to form calcium deposits. This was also confirmed by gene profile of osteogenic markers ALP, osteopontin, and osteocalcin. Thus, cells harvested from the trabecular bone of femoral head and neck possess a higher intrinsic osteogenic potential compared with cells isolated from the acetabular subchondral bone.

We also proved that an inflammatory environment, obtained by supplementing the osteogenic medium with IL1-*β* at low dose (50 pg/ml), did not affect the osteogenic differentiation. Moreover, our results suggest that a controlled inflammatory stimulus may promote the osteogenic potential of multipotent cells without leading to cell death or tissue damage.

In summary, our study analyzed and compared the multipotency of cells derived from different anatomical sites during hip replacement surgery: the bone marrow from the femoral canal, pieces of subchondral acetabular bone, and fragments of bone from femoral neck and head. Our data confirmed that BMCs are the most versatile multipotent cells as they display a similar adipogenic, chondrogenic, and osteogenic potential. In addition, we demonstrated that FC possess a significantly higher osteogenic potential compared to AC. Thus, these cells are probably precommitted to the osteogenic lineage. This novel finding may be important for the development of new techniques of bone stock restoration and also new strategies of bone tissue engineering. Indeed, the intrinsic osteogenic precommitment of these cells may potentially allow for a faster and better bone repair.

In conclusion, to answer our initial question, the harvesting site does influence the osteogenic potential of multipotent cells in an *in vitro* setting. *In vivo* studies will be necessary to confirm our preliminary results.

## Figures and Tables

**Table 1:** Primer sequences for qPCR (F/R: forward/reverse).

	Human gene	Sequence (5′-3′)	Product size (bp)
Housekeeping genes	18S rRNA	F: GTAACCCGTTGAACCCCATT	151 [21]
		R: CCATCCAATCGGTAGTAGCG	
	GAPDH (NM_001289745.2)	F: ATGGGGAAGGTGAAGGTCG	70
		R: TAAAAGCAGCCCTGGTGACC	

Adipogenic differentiation	PPARG (NM_001354667)	F: GGCTTCATGACAAGGGAGTTTC	74
		R: AACTCAAACTTGGGCTCCATAAAG	
	LPL (NM_000237.2)	F:GAGGTACTTTTCAGCCAGGATGTAAC	82
		R: AGCTGGTCCACATCTCCAAGTC	

Chondrogenic differentiation	COL1A1 (NM_000088.3)	F: CAGCCGCTTCACCTACAGC	83
		R: TTTTGTATTCAATCACTGTCTTGCC	
	COL2A1 (NM_001844.5)	F: GGCAATAGCAGGTTCACGTACA	79
		R: CGATAACAGTCTTGCCCCACTT	
	Sox9 (NM_000346.3)	F: AGTACCCGCACTTGCACAA	68
		R: CTCGTTCAGAAGTCTCCAGAGCTT	

Osteogenic differentiation	BGLAP (osteocalcin) (NM_199173.5)	F: CGCCTGGGTCTCTTCACTAC	143
		R: CTCACACTCCTCGCCCTATT	
	SPP1 (osteopontin) (NM_001251830.1)	F: CTCAGGCCAGTTGCAGCC	81
		R: CAAAAGCAAATCACTGCAATTCTC	
	ALP (NM_000478.6)	F: ACCACCACGAGAGTGAACCA	79
		R: CGTTGTCTGAGTACCAGTCCC	

18S rRNA: 18S ribosomal RNA; GAPDH: glyceraldehyde-3-phosphate dehydrogenase; PPARG: peroxisome proliferator-activated receptor gamma; LPL: lipoprotein lipase; COL1A1: collagen type I alpha 1 chain; COL2A1: collagen type II alpha 1 chain; BGLAP: bone gamma-carboxyglutamate protein; SPP1: secreted phosphoprotein 1; ALP: alkaline phosphatase.

## Data Availability

The GraphPad Prism files and all the cytometry data used to support the findings of this study are available from the corresponding author upon request.

## Additional Points

The project number L1033 is registered with ClinicalTrials.gov NCT03369457.
